# Supplementary material for: The dynamics of protein localisation to restricted zones within Drosophila mechanosensory cilia
Source: Sci Rep. 2022 Aug 3;12:13338. doi: 10.1038/s41598-022-17189-w (PMC9349282; doi:10.1038/s41598-022-17189-w)
Supplement: Supplementary file 1 — Supplementary Information. [file 41598_2022_17189_MOESM1_ESM.pdf]

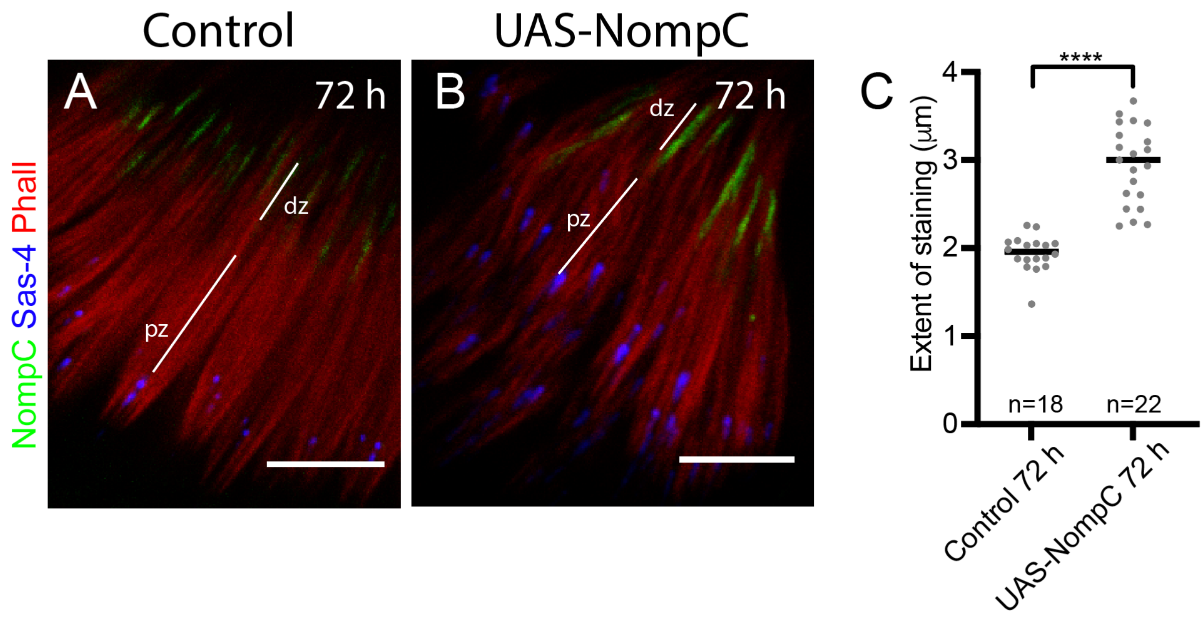

Supplementary Fig. S1. NompC overexpression does not strongly alter its localisation to the distal zone

(A,B) 72-h pupal antenna showing NompC (green), Sas-4 (blue), phalloidin (red). (A) Control (*scaGal4*/+). (B) NompC overexpression (*scaGal4*, UAS-*NompC*), showing longer extent of NompC labelling. (C) Graph comparing length of NompC labelling. Significance determined by two-tailed Mann-Whitney U test (****: P<0.0001). All scale bars are 5 µm.

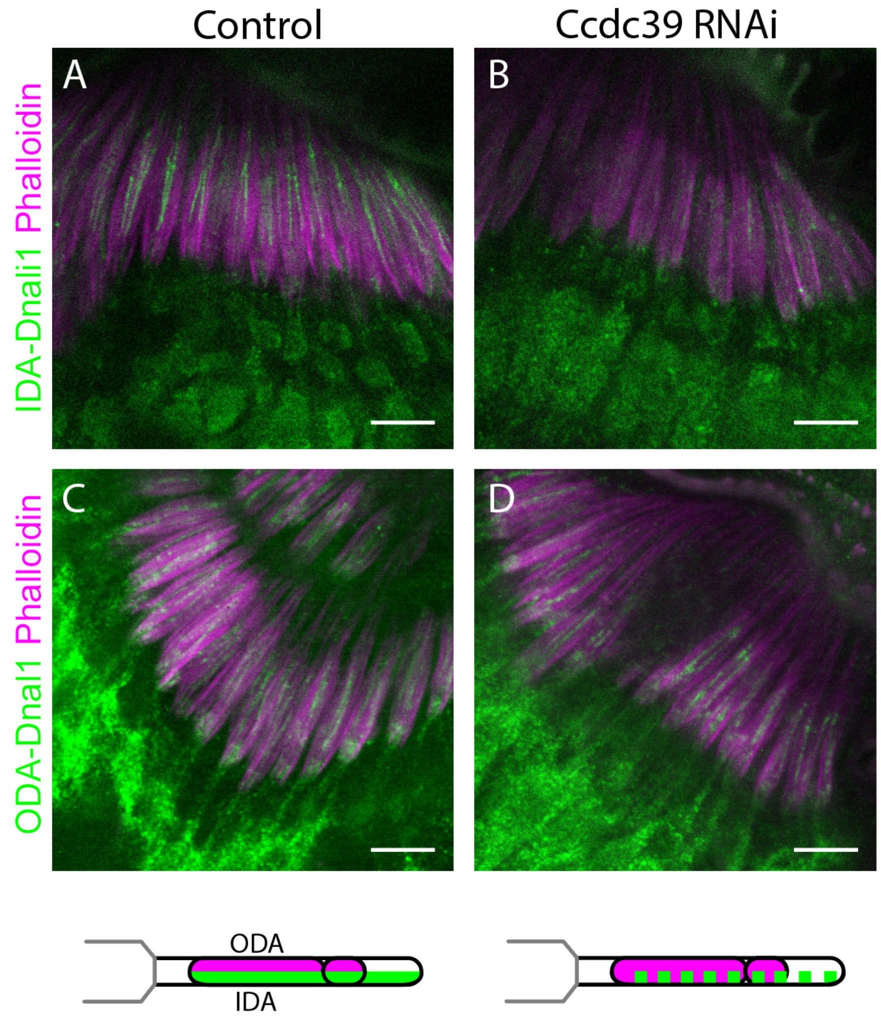

Supplementary Fig. S2. Knockdown of *Ccdc39* reduces localisation in the cilium IDA-Dnali1 but not ODA-Dnal1.

72-h pupal antennae. (A,B) Immunofluoresence for IDA-Dnali1-mVenus (green), phalloidin (magenta). (A) Control (*scaGal4*/+). (B) Ccdc39 knockdown (*scaGal4*, UAS-*Ccdc39*-RNAi). (C,D) Immunofluoresence for ODA-Dnal1-mVenus (green), phalloidin (magenta). (C) Control (*scaGal4*/+). (D) Ccdc39 knockdown (*scaGal4*, UAS-*Ccdc39*-RNAi). Below the panels is a schematic summary. All scale bars are 5 μm.

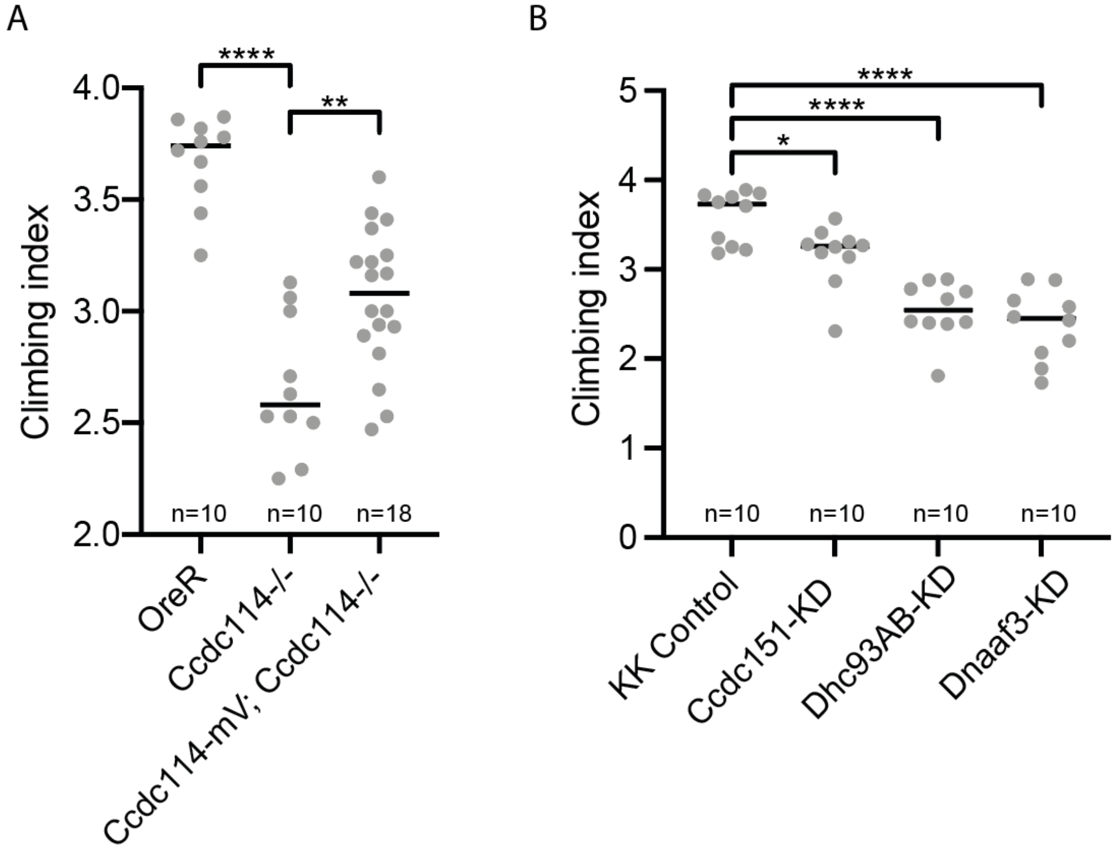

Supplementary Fig. S3. Climbing assays to test proprioceptive behaviour of flies

(A) *Ccdc114* homozygote flies perform significantly worse than wild-type flies (OreR) in a climbing assay (****: P<0.0001). Rescue of homozygotes by one copy of the Ccdc114-mVenus fusion gene improved their performance significantly compared to homozygotes (**: P=0.0027). Significance was determined by one-way Anova followed by Dunnett's test for multiple comparisons. n = 10 batches of 15 flies (or 18 batches for rescue cross). (B) Knockdown of *Ccdc151* and *Dhc93AB* (*scaGal4*, UAS-RNAi) result in impaired performance in climbing assay compared to control (*scaGal4*, KK control line). For comparison, knockdown of *Dnaaf3* is also included, in which dynein arms are completely absent from the cilia (zur Lage et al., 2021). Significance was determined by one-way Anova followed by Dunnett's test for multiple comparisons: *CG14127*: p = 0.0246; *Dhc93AB*: P < 0.0001; *Dnaaf3*: P<0.0001. n = 10 batches of 15 flies.

Supplementary Table S1. Oligonucleotides used in this study

Name of oligonucleotide	Sequence
**mVenus constructs**	
CG6971-L	GGGGACAAGTTTGTACAAAAAAGCAGGCTG AATTGGAAATGTGCAACGA
CG6971-R	GGGGACCACTTTGTACAAGAAAGCTGGGTC CTTCTTGGGTGCGGTTATGC
CG8800-attB Fw	GGGGACAAGTTTGTACAAAAAAGCAGGCTG GCAAGCGTGGAAATCGCAAG
CG8800-attB Rv	GGGGACCACTTTGTACAAGAAAGCTGGGTC AAGCTGCGGCTCCTCGTTGAGC
CG17387mVenus left	GGGGACAAGTTTGTACAAAAAAGCAGGCTT GACTACCGGCTTTGAGAAAT
CG17387mVenus right	GGGGACCACTTTGTACAAGAAAGCTGGGTC TTTTTTCTTCGGCAAGGGCAACTC
CG14905mVenus 5	GGGGACAAGTTTGTACAAAAAAGCAGGCTG TACAAATCGAGCGCTGCGTTGCTC
CG14905mVenus 3	GGGGACCACTTTGTACAAGAAAGCTGGGTC GTTTTTAGTTTTCGCCGATTCCCGT
**pBID modification**	
pBID UAS_del	TCGATCCGCTTGCATGCCTGCAGGTATCGAGCGCAGCGGTATAAAAGGGC
**Gibson cloning of pBID-GmCherry**	
cassette-fwd	CGCAGATCTGCGGCCGCGGCTCGAGGGCCGGATCCGAA
cassette_rev	TGCTCACGCATGCGATATCAACCACTTTGTACAAGAAAGC
mCherry_fwd	TTGATATCGCATGCGTGAGCAAGGGCGAG
mCherry_rev	GGTTCCTTCACAAAGATCCTCTAGACTACTTGTACAGCTCGTCC
**CG14905 CRISPR RNA guides**	
RNA guide oligonucleotide 1 sense	CTTCGGTGGCAAGTTGCTTAGTAA
RNA guide oligonucleotide 1 antisense	AAACTTACTAAGCAACTTGCCACC
RNA guide oligonucleotide 2 sense	CTTCGTGTTCCCAAATTCAACATA
RNA guide oligonucleotide 2 antisense	AAACTATGTTGAATTTGGGAACAC
**CG14905 CRISPR homology arms**	
Left HA EcoRI 14905	GCCGAATTCGCAACAACAACCACAACAGC
Left HA NotI 14905	GATGCGGCCGCGGTTTATCGAAGACTTTTACC
right HA PstI 14905	GCCCTGCAGTCGGCGAAAACTAAAAACTA
Right HA XhoI 14905	GATCTCGAGGGCAGAAGGACCCAGTAAGT
